# Noninvasive brain oxygen saturation measurement during caloric response in vertigo patients—preliminary report

**DOI:** 10.3389/fneur.2025.1556265

**Published:** 2025-06-16

**Authors:** Magdalena Nowaczewska, Maria Marzec, Łukasz Kluczyński, Katarzyna Sierakowska, Maciej Wróbel

**Affiliations:** ^1^Department of Otolaryngology, Head and Neck Surgery, and Laryngological Oncology, Ludwik Rydygier Collegium Medicum in Bydgoszcz, Nicolaus Copernicus University, Bydgoszcz, Poland; ^2^Polyclinic, Laryngology Outpatient Clinic, Oncology Center Prof. F. Łukaszczyk in Bydgoszcz, Bydgoszcz, Poland; ^3^Department of Human Physiology, Collegium Medicum, Nicolaus Copernicus University, Bydgoszcz, Poland; ^4^Faculty of Medicine, Bydgoszcz University of Science and Technology, Bydgoszcz, Poland

**Keywords:** caloric test, brain oximetry, videonystagmography, vertigo, oxygenation

## Abstract

**Introduction:**

Although videonystagmography (VNG) is a useful test for evaluating patients with vestibular symptoms, it may cause undesirable side effects. The aim of this study was to measure cerebral tissue oxygen saturation in patients with vertigo undergoing VNG and assess its relationship with clinical parameters, including the severity of the procedure-related side effects.

**Materials and methods:**

Continuous measurement of regional oxygen saturation (rSO_2_) from both hemispheres during VNG with caloric stimulation was performed in patients with chronic vertigo using a near-infrared spectroscopy sensor. This sensor, connected to a regional oximetry system (O3™, Masimo, Irvine, CA), was placed on the participant’s forehead. During and after the VNG procedure, patient-perceived dizziness, discomfort, headache, and nausea—side effects related to VNG testing—were assessed using a 0-to-10 visual analog scale (VAS).

**Results:**

A total of 23 patients were enrolled, with a mean age of 54.27 years and an average duration of vertigo of 5.96 years. Of these, 11 patients experienced VNG-related side effects. No significant differences in oximetry parameters were observed before and after the caloric test, regardless of whether cold or hot water was used or whether the left or right ear was stimulated. We found a positive correlation between the overall change in oxygenation values in both hemispheres during the whole VNG test (delta) and the severity of side effects symptoms (VAS). The correlation coefficient between delta and VAS was higher in the right hemisphere than in the left (accordingly 0.69 vs. 0.62, *p* < 0.05).

**Conclusion:**

Caloric stimulation does not influence regional brain oxygenation as measured by a regional oximetry system. However, fluctuations in rSO_2_ values during the whole VNG procedure, predominantly on the right side, may be associated with the side effects of the procedure.

## 1 Introduction

Videonystagmography (VNG) is a common test used to evaluate patients with vestibular symptoms. The caloric test with induced nystagmus registration is a frequently used diagnostic tool to estimate labyrinth function (1). Although this examination can help establish baseline vestibular function in patients with vertigo, it has certain limitations such as undesirable patient symptoms during testing and post-VNG morbidity (1). Kelly et al. discovered that approximately 58% of patients experienced side effects during testing, including nausea, vomiting, and headaches. In addition, 15% discontinued testing early due to the side effects (2).

In the caloric test, heat transfer from the external auditory meatus to the inner ear results in endolymph movement and alters the bioelectric potential of the stimulated semicircular canal. This leads to neuronal depolarization and, ultimately, increased conduction along cranial nerve VIII. Low temperature causes the endolymph to move in the opposite direction with respect to hot stimulation. Then, nerve VIII conduction is reduced compared to the zero state. The induced stimulus activates the vestibulo-cerebello-oculomotor (vestibulo-oculomotor) and vestibulo-cerebello-spinal reflexes. This may potentially affect cerebral blood flow or cerebral oxygenation, which may be linked, in some patients, to unpleasant side effects (1–3).

Nowadays, modern near-infrared spectroscopy technology is increasingly used to measure cerebral tissue oxygen saturation (4). Nevertheless, there is a lack of studies assessing brain oxygen saturation during VNG tests.

The aim of this study was to measure cerebral tissue oxygen saturation using the novel O3 Regional Oximetry^®^ device (Masimo Corporation, Irvine, CA, USA) in patients with vertigo undergoing VNG and assess its relationship with clinical parameters, including the severity of the procedure-related side effects.

## 2 Materials and methods

This prospective study involved consecutive patients with chronic vertigo who had undergone vestibular assessment at our ENT department between April 2024 and August 2024. Patients were included in this study if they had vertigo or dizziness for at least 3 months and were eligible to undergo VNG testing. Patients with incomplete caloric testing, congenital or periodic alternating nystagmus, unilateral or bilateral vestibulopathy, internal carotid artery stenosis, or signs of brain damage on MRI were excluded. During the baseline visit, all patients underwent detailed history taking and clinical evaluations according to the standard protocol developed in our clinic. Data on age at vertigo onset, vertigo type, presence of hearing loss, headache, and other comorbidities were also collected. All patients were right-handed.

The typical VNG test battery was performed using the Synapsys VNG modular system and included the following: ocular motor tasks [sitting with gaze holding at center, right, left, up, and down (with/without fixation)], saccades, anti-saccades, pursuit, and optokinetics; head shake tests (horizontal and vertical, 20 s each); positional testing including the Dix–Hallpike and roll tests; positional testing with fixation denied in supine, body right, and body left positions; precaloric testing; and caloric evaluation performed in a 30-degree supine body position with bithermal water irrigations for 30 s (warm 44°C and cool 30°C).

During and after the VNG procedure, patient-perceived dizziness, discomfort, headache, and nausea—side effects related to VNG testing—were assessed using a 0-to-10 visual analog scale (VAS), where 0 indicated no symptoms and 10 represented the worst possible severity. All our patients also underwent the vHIT test along with the other vestibular tests. However, we did not include the vHIT in our study because during the rapid, high-velocity, low-amplitude, and unexpected head movements, oxygenation was not accurately measured, generating incorrect results.

### 2.1 Oxygenation

The patient’s oxygenation status was assessed using Root with O3 Regional Oximetry and Masimo SET^®^ Pulse Oximetry (SpO2) O3. SctO_2_ was recorded using the O3-RO^®^ system. This was achieved through the placement of a disposable adhesive sensor on each side of the forehead. The sensor contains one light source and two photodetectors: intracranial and extracranial. Infrared light is emitted and reflected toward both detectors. The intracranial detector measures hemoglobin oxygen saturation in the outer frontal cerebral cortex at a depth of 20 mm. The shallower extracranial detector measures superficial tissues that may be affected by photon scatter as a result of extracerebral tissue reflections, significant variations in pigmentation, and brain water content (4). The pictures of the sensors and the device are included in Supplementary materials.

Oxygenation (rSO_2_) was continuously recorded during the whole VNG procedure in both the left and right hemispheres. Then, we extracted the results before each caloric irrigation (baseline) and during the peak of the nystagmic response (peak). The conditions are abbreviated as follows: LC for left cool, LW for left warm, RC for right cool, and RW for right warm. We also assessed fluctuations in oxygenation in the right and left hemispheres during the whole procedure. We measured the overall change in oxygenation values during the whole VNG test (delta) in both hemispheres and correlated it with the severity of side effects symptoms. rSO_2__Delta was calculated as the absolute difference between baseline and peak rSO_2_ values in the right and left hemispheres.

Our study was approved by the Local Ethics Committee of the Ludwik Rydygier Collegium Medicum in Bydgoszcz. Specific written consent was required for this prospective study.

### 2.2 Statistics

The data were tested for normality using the Shapiro–Wilk test. Then, non-parametric tests were used to compare continuous variables between the two groups. To assess the oximetry changes during the caloric test, the baseline and peak values were compared using the Wilcoxon signed-rank test. To assess the relationship between individual oximetry parameters, the Pearson correlation coefficient was used. Friedman’s ANOVA was used to analyze the oximetry results for every stage of the caloric test. The Mann–Whitney U test was performed to compare the oximetry results between the left and right hemispheres for continuous independent variables. For all analyses, the level of statistical significance was set at a *p*-value of 0.05. All calculations were performed using *Statistica 13*.

## 3 Results

A total of 29 patients were enrolled; however, three discontinued VNG early due to vomiting and were therefore excluded from the study. The mean age was 54.27 years, and the average duration of vertigo was 5.96 years. In addition, 11 (42.31%) patients experienced VNG-related side effects.

The clinical characteristics of the patients are presented in Table 1.

**Table 1:** The clinical characteristics of the patients.

Variable	Parameter	Group (*N* = 26)
Sex	Female	84.6% (*N* = 22)
	Male	15.4% (*N* = 4)
Age [years]	Mean (SD)	54.3(10.9)
Duration of vertigo [years]	Mean (SD)	6 (9.4)
Diagnosis	Vestibular migraine	19.2% (*N* = 5)
	Vestibular paroxsyzmia	11.5% (*N* = 3)
	BPPV	7.7% (*N* = 2)
	Meniere’s disease	7.7% (*N* = 2)
	PPPD	7.7% *N* = 2
	Other	4.2% (*N* = 12)
Hearing loss	Yes	42.3% (*N* = 11)
Depression and anxiety	Yes	30.8% (*N* = 8)
Headache	Yes	46.2% (*N* = 12)
Tinnitus	Yes	42.3% (*N* = 11)
Hashimoto	Yes	11.5% (*N* = 3)
VNG-related side effects	Yes	42.3%(*N* = 11)

After comparing the oximetry parameters before and after the caloric test, we did not find any significant differences related to the temperature of the water (cold or hot) or the side tested (left or right ear) (Table 2). There were also no significant differences in peripheral oxygen saturation, mean blood pressure, and heart rate before and after the caloric test and during the whole VNG procedure.

**Table 2:** Regional oxygen saturation (rSO_2_) before bilateral caloric irrigation (baseline) and at the peak of the nystagmic response (peak).

Parameter		Mean	SD	Min	Max	*P*-value
rSO_2_, right hemisphere [%]	Baseline RC	65.19	4.92	56.00	77.00	0.66
	Peak RC	65.15	4.91	56.00	77.00	
	Baseline LC	65.23	4.48	57.00	77.00	0.46
	Peak LC	64.81	5.26	54.00	77.00	
	Baseline RW	64.46	4.54	58.00	76.00	
	Peak RW	65.12	5.30	56.00	79.00	0.05
	Baseline LW	63.96	5.55	51.00	77.00	0.21
	Peak LW	64.73	4.42	58.00	77.00	
rSO_2_, left hemisphere [%]	Baseline RC	64.50	4.09	57.00	73.00	0.22
	Peak RC	64.92	3.68	59.00	73.00	
	Baseline LC	64.77	4.00	58.00	73.00	0.60
	Peak LC	64.69	4.29	57.00	74.00	
	Baseline RW	64.69	3.98	58.00	73.00	0.72
	Peak RW	64.58	4.08	56.00	73.00	
	Baseline LW	64.46	3.93	58.00	73.00	1.00
	Peak LW	64.46	3.98	56.00	73.00	

LC, left cool; LW, left warm; RC, right cool; RW, right warm.

**Figure 1:**
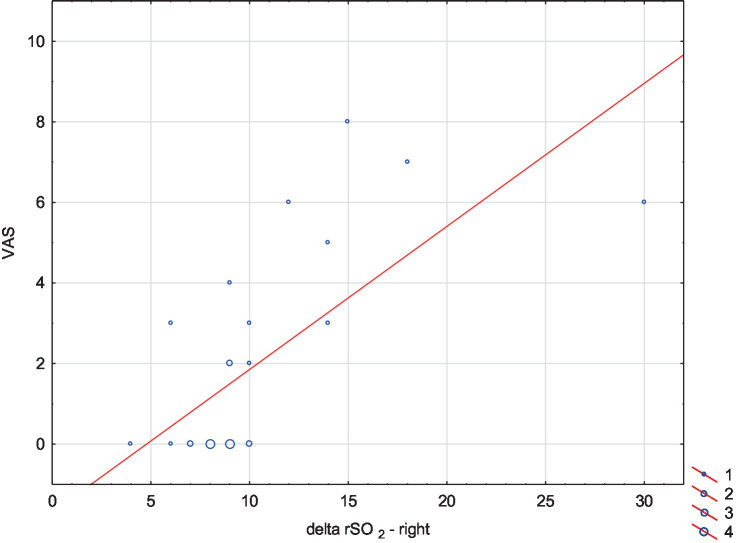
Correlation plot showing the relationship between the overall change in oxygenation values in the right hemisphere during the whole VNG test (delta rSO_2_ left) and the severity of side effects symptoms (VAS). A positive correlation was observed between VAS scores and delta rSO_2_ in the right hemisphere (correlation coefficient = 0.69, *p* < 0.05).

**Figure 2:**
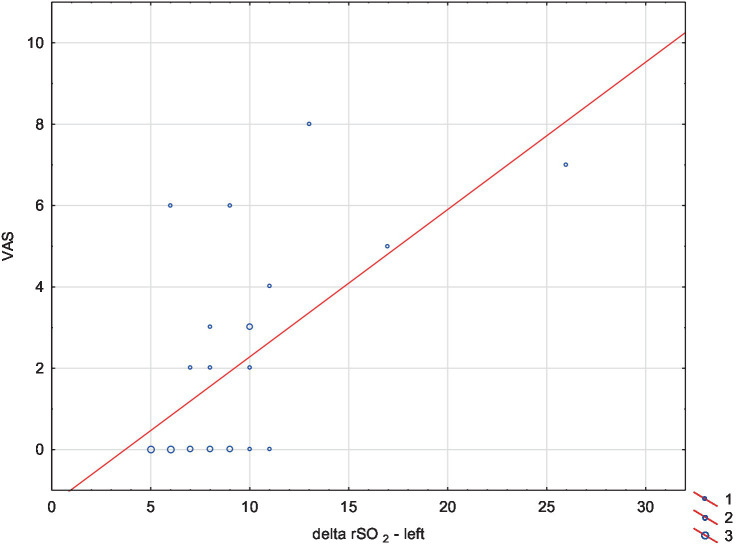
Correlation plot showing the relationship between the overall change in oxygenation values in the left hemisphere during the whole VNG test (delta rSO_2_ left) and the severity of side effects symptoms (VAS). A positive correlation was observed between VAS values and delta rSO_2_ in the left hemisphere (correlation coefficient = 0.62, *p* < 0.05).

After comparing rSO_2__delta between the right and left hemispheres, we did not find any significant differences. We found a positive correlation between the overall change in oxygenation values during the whole VNG test (rSO_2__delta) in both hemispheres and the severity of side effects symptoms (VAS). The correlation coefficient between VAS scores and rSO_2__delta in the right hemisphere was higher than in the left hemisphere (accordingly 0.69 vs. 0.62, *p* < 0.05) (Table 3, Figures 1, 2).

**Table 3:** Regional oxygen saturation (rSO_2_) values at the beginning of the VNG procedure (baseline) and at the peak of the nystagmic response (max) and the overall change in rSO_2_ during the whole VNG procedure (delta).

Parameter	*n*	Mean	Median	Min.	Max	SD
rSO_2_ Baseline L [%]	26	64.26	64	58	71	3.65
rSO_2_ Baseline R [%]	26	65.15	65	56	72	4.23
rSO_2_ max RHLC [%]	26	64.92	64	59	73	3.68
rSO_2_ max LHLC [%]	26	64.69	64	57	74	4.29
rSO_2_ max RHRC [%]	26	65.15	65	56	77	4.91
rSO_2_ max LHRC [%]	26	64.81	65.5	54	77	5.26
rSO_2_ DELTA L [%]	26	9.11	8	5	26	4.38
rSO_2_ DELTA R [%]	26	10.31	9	4	30	5.03
VAS	26	1.96	0	0	8	2.57

LC, left cool; RC, right cool; RH, right hemisphere; LH, left hemisphere.

## 4 Discussion

To the best of our knowledge, this is the first study to investigate direct cerebral tissue oxygen saturation using a regional oximetry system in patients undergoing VNG with the caloric test.

Our hypothesis was that caloric stimulation may influence brain oxygenation. To date, several fMRI (functional near-infrared spectroscopy) and PET studies have identified central and cortical brain regions involved in the vestibular network (5). Activation of specific areas—including the posterior insula and retroinsular regions, the superior temporal gyrus (STG), and the inferior parietal lobule (IPL)—has been observed during caloric irrigation in neuroimaging studies (5). Wypych et al. studied the pattern of grey matter local activation using fMRI during cold and hot caloric stimulation in patients with unilateral vestibular injury and observed increased activity in multiple areas, such as the right frontoparieto-temporal cortex, hippocampus, and cerebellar hemispheres (6). Iida et al. recorded fNIRS signals from the temporoparietal region of the brain in five healthy volunteers during unilateral warm and cool caloric irrigations. During warm-water stimulation, they observed increased bilateral blood volume changes, with larger changes in the hemisphere ipsilateral to the side of stimulation, while during cool-water stimulation, they observed bilateral decreases in total hemoglobin (7). In contrast to previous studies, we found no differences in the oxygenation value before and after the caloric test and no difference in the overall change in the oxygenation value during the whole VNG test (delta) in both hemispheres. One reason for this may be the device we used to measure oxygenation. The O3™ device measures regional tissue oxygen saturation, while fMRI detects changes in cerebral blood oxygenation. Another reason may be the small number of participants, as well as the highly heterogeneous group. The most important finding of our study is the positive correlation between the overall change in oxygenation values during the whole VNG test (delta rSO_2_) in both hemispheres and the severity of side effects symptoms (measured by the VAS). This means that the patients with more severe side effects experienced greater fluctuations in oxygenation values during the whole VNG procedure. We also found that the correlation between delta rSO_2_ and side effect severity was stronger on the right side. Similarly, Wypych et al. revealed that more brain centers were stimulated by the cold stimulus applied to the right ear compared to the left ear and also that the differences between the hot and cold stimuli were more pronounced for the right ear (6). Karim et al. found that cool water irrigation produced dominant activation contralateral to the stimulated ear. However, warm caloric stimulation produced more bilateral activation than the cool stimulus, with generally greater activation in the right hemisphere (8).

Fasold et al. demonstrated a strong right-hemispheric dominance of vestibular cortex areas regardless of the stimulated side, consistent with the current understanding of a rightward asymmetrical cortical network for spatial orientation (9). According to some authors, the changes in cortical brain activity are not a direct result of the sensation of the caloric irrigation or an artifact of the device itself but are instead related, either directly or indirectly, to the underlying physiological vestibulo-ocular response (8). The question arises: why do individuals experience fluctuations in oxygenation? According to several authors, spontaneous fluctuations may be produced when cerebral perfusion is challenged by systemic or local manipulations. The most potent stimuli are hypotension, hyperventilation, cerebral artery occlusion, and cerebral vasoconstriction (10). In our patient group, blood pressure and peripheral oxygenation were stable, while other factors were less probable. Therefore, the change in oxygenation might be a result of the VNG procedure itself, probably due to visual stimulation.

Our study revealed that approximately 42% of the patients experienced VNG-related side effects. Kelly et al. found that 58% of patients experienced specific complications during testing, with greater complication rates observed in patients with a self-reported history of headaches. Mean total eye speed during caloric irrigations was higher in patients who experienced complications compared to those who did not (2). Contrary to their results, our group with VNG complications did not show a higher prevalence of headache history. It has also been demonstrated that the caloric test may provoke emotional reactions and stress (11).

There are several limitations in our study. First, the small number of participants and the absence of a control group. In addition, it should be noted that Masimo SET^®^ Pulse Oximetry (SpO2) detectors measure hemoglobin oxygen saturation in the outer frontal cerebral cortex at a depth of 20 mm, which is not the only brain area involved in the vestibular response. Another limitation may be the heterogeneous group of patients with different vertigo etiologies.

## 5 Conclusion

Our preliminary report suggests that caloric stimulation does not influence regional brain oxygenation as measured by a regional oximetry system. However, fluctuations in rSO_2_ values during the whole VNG procedure, predominantly on the right side, may be associated with the side effects of the procedure.

## Data Availability

The raw data supporting the conclusions of this article will be made available by the authors, without undue reservation.
