# Supplementary material for: Noninvasive brain oxygen saturation measurement during caloric response in vertigo patients—preliminary report
Source: Front Neurol. 2025 Jun 16;16:1556265. doi: 10.3389/fneur.2025.1556265 (PMC12206696; doi:10.3389/fneur.2025.1556265)
Supplement: Supplementary file 1 [file Supplementary_file_1.docx]

Supplementary materials

1. Photos of sensors placed on the forehead together with VNG goggles, as well as description of the procedure and measurements.

*
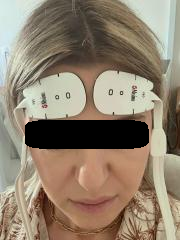
***
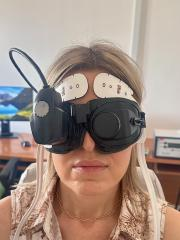

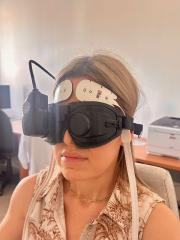
**

**
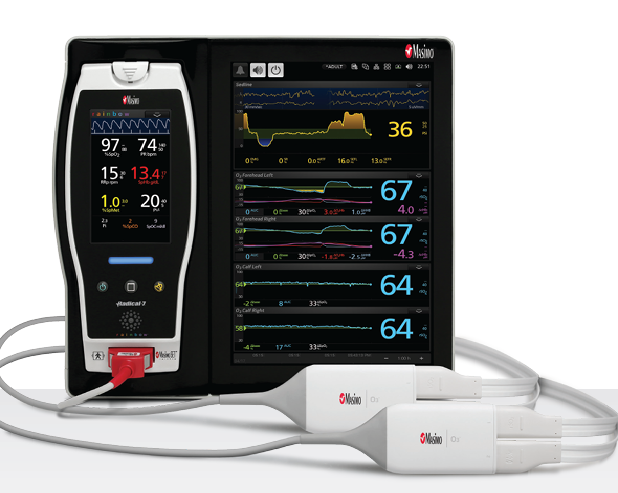
**

O3 Regional Oximetry monitors the regional hemoglobin oxygen saturation of blood (rSO2) in the region of interest. With their flexible design, O3 sensors easily conform to and allow for ergonomic application.

Regional Oxygenation Information:

1. Regional Oxygenation (rSO2) rSO2, measured in percentage (0 to 99%), is the regional tissue oxygenation level in the deep tissue local to the sensor site.
2. AUC Index (Area under the Curve) AUC, measured in % * minutes, is displayed as an index which quantifies the duration and depth of patient’s stay below the user-defined rSO2 low alarm limit
3. Baseline rSO2 displays the user-defined baseline value for rSO2. Baseline is also shown as a triangular pointer on the y-axis of the trend display for rSO2
4. Delta Baseline (Δbase) measured in percentage, is the relative decrease in rSO2 with respect to baseline rSO2.
5. Operation Delta SpO2 (ΔSpO2) ΔSpO2, displayed as a percentage, is the calculated difference between rSO2 and SpO2. Source of SpO2 is from peripheral SpO2 (using pulse oximeter, if available), depending on user selection.
